# Role of Exogenous and Endogenous Hydrogen Sulfide (H_2_S) on Functional Traits of Plants Under Heavy Metal Stresses: A Recent Perspective

**DOI:** 10.3389/fpls.2020.545453

**Published:** 2021-01-07

**Authors:** Muhammad Saleem Arif, Tahira Yasmeen, Zohaib Abbas, Shafaqat Ali, Muhammad Rizwan, Nada H. Aljarba, Saad Alkahtani, Mohamed M. Abdel-Daim

**Affiliations:** ^1^Department of Environmental Science and Engineering, Government College University Faisalabad, Faisalabad, Pakistan; ^2^Department of Biological Sciences and Technology, China Medical University, Taichung, Taiwan; ^3^Department of Biology, College of Science, Princess Nourah Bint Abdulrahman University, Riyadh, Saudi Arabia; ^4^Department of Zoology, College of Science, King Saud University, Riyadh, Saudi Arabia; ^5^Pharmacology Department, Faculty of Veterinary Medicine, Suez Canal University, Ismailia, Egypt

**Keywords:** biochemical properties, physiological activities, heavy metal stress, hydrogen sulfide, signaling molecule, oxidative impairment

## Abstract

Improving growth and productivity of plants that are vulnerable to environmental stresses, such as heavy metals, is of significant importance for meeting global food and energy demands. Because heavy metal toxicity not only causes impaired plant growth, it has also posed many concerns related to human well-being, so mitigation of heavy metal pollution is a necessary priority for a cleaner environment and healthier world. Hydrogen sulfide (H_2_S), a gaseous signaling molecule, is involved in metal-related oxidative stress mitigation and increased stress tolerance in plants. It performs multifunctional roles in plant growth regulation while reducing the adverse effects of abiotic stress. Most effective function of H_2_S in plants is to eliminate metal-related oxidative toxicity by regulating several key physiobiochemical processes. Soil pollution by heavy metals presents significant environmental challenge due to the absence of vegetation cover and the resulting depletion of key soil functions. However, the use of stress alleviators, such as H_2_S, along with suitable crop plants, has considerable potential for an effective management of these contaminated soils. Overall, the present review examines the imperative role of exogenous application of different H_2_S donors in reducing HMs toxicity, by promoting plant growth, stabilizing their physiobiochemical processes, and upregulating antioxidative metabolic activities. In addition, crosstalk of different growth regulators with endogenous H_2_S and their contribution to the mitigation of metal phytotoxicity have also been explored.

## Introduction

Heavy metals are a group of metal elements having peculiar physical and chemical properties, which are also known to possess higher specific gravity > 4 g cm^–3^ in nature (Grant and Grant, 1987; Duffus, 2002). Environmental occurrence of heavy metal can be of both natural and anthropogenic origin; however, unprecedented release and their strong ecological persistence have now become a serious toxicological and public health challenge worldwide (Arif et al., 2019; Zheng et al., 2020). Under natural conditions, heavy metals are the intrinsic component of earth crust and are often dispersed in soil, water, and atmosphere as a result of many geological processes, i.e., forest fire and volcanic eruption (Lado et al., 2008). A range of anthropogenic activities, such as intensive pesticides, as well as fertilizers use, vehicular emissions, mining activities, and industrial wastes, are the main contributors of heavy metal pollution across various domains of environment. Besides this, heavy environmental loading of metal toxicants also emanates from different wastewater sources. Globally, large volume of untreated wastewater is being discharged directly into waterways and soil, where they pose serious concerns for ecosystem stability (Mataka et al., 2006).

In soil, level of individual heavy metal concentration is a primary indicator often used to determine the degree of ecotoxicological effects on plants. For instance, some metals, such as nickel (Ni), molybdenum (Mo), zinc (Zn), and copper (Cu), are plant micronutrients and are phytotoxic only if their concentration is higher in soil (Lasat, 2002), whereas few other metallic elements, in particular chromium (Cr), lead (Pb), and cadmium (Cd), are hazardous to plants even at low soil concentrations. Heavy metals can either be found in dissolved or immobilized form; however, high immobilization rates can cause stronger detrimental effects on plants because of their *in situ* persistence and concentration buildup over time (Alloway, 1995, 2013). Consequently, they tend to impair various growth attributes of plants (Ahmad et al., 2012). Furthermore, some of these metals, such as Cr, Cd, Mn, and Zn, are also recognized to exert hormetic responses, which are reflected by a positive growth response at low concentrations and by phytotoxicity at higher metal concentrations (Azevedo and Lea, 2005). Detrimental effects of heavy metals on plants may involve oxidative stress, stunted growth, and toxicity-induced metabolic anomalies.

After nitric oxide and carbon monoxide, hydrogen sulfide (H_2_S) is the third most important naturally occurring gaseous molecule known for its cellular signaling in biology (Yang et al., 2008). In plants, synthesis and release of H_2_S typically occur during different stages of metabolic activities. It is generally formed in cut branches, tissue cultures, and leaf discs, whereas it is discharged into the surrounding environment from green cells of the plants (Rennenberg et al., 1990). Under normal growth conditions, numerous plants such as soybean, pumpkin, cotton, cantaloupe, squash, corn, and cucumber were found to release H_2_S from leaf into the exterior environment (Wilson et al., 1978). Besides increasing enzymatic activity of vigorous cysteine (Cys), H_2_S secretion is also recognized to enhance sulfite and sulfate metabolic activities (Rennenberg, 1983, 1984). However, numerous reports have observed paradoxical functions of H_2_S in the regulation of plants physiological and biochemical traits. For instance, H_2_S has been established to act as sulfur source at lower concentration, while promoting phytotoxic effects on plant growth at elevated level (De Kok et al., 2002; Li, 2013; Hancock and Whiteman, 2016; Li et al., 2016; Hancock, 2017; Huo et al., 2018). In most plants, elongated exposure to higher H_2_S level eventually caused leaf removal after developing leaf injury, which can lead to overall deterioration of plant growth. Likewise, H_2_S triggered O_2_ obstruction and subsequent impaired nutrient acquisition in rice seedlings were reported by Wang et al. (2012). Positive influence of H_2_S on various plants has also been reported in literature. Shoot deposited sulfur (S) from H_2_S appears to provide major site-specific S regulation for plants to improve plant growth, particularly under sulfur-deprived conditions. In many plants, H_2_S is reported to be involved in the regulation of key physiological and growth functions, such as formation of adventitious root in cucumber, stomatal conductance in *Arabidopsis thaliana*, enhanced tolerance against salinity in alfalfa during seed germination, and regulation of thiol levels in *A. thaliana* (Riemenschneider et al., 2005a,b; Lisjak et al., 2010; Lin et al., 2012).

The H_2_S is a convenient stress signaling molecule, as its biosynthesis process can take place in various cellular components once plants experience stress such as heavy metal exposure (Zulfiqar and Hancock, 2020). Upon its production, H_2_S can be highly mobile across plant membranes and can either be influxed in or effluxed out of the plant system as a way out against heavy metal stress (Shivaraj et al., 2020). Plant stress adaptation against heavy metal in H_2_S-treated plant is initiated via antioxidant activities (Kushwaha and Singh, 2019), accumulation of osmoregulators (Tian et al., 2016), cell signaling protein (He et al., 2019), and by different gene expressions (Pandey and Gautam, 2019). Overall, it enables plants to combat against stress factor such as heavy metals via effective removal of reactive oxygen species (ROS) by adjusting intracellular redox balance.

A plant experiment with nickel spiking has demonstrated that H_2_S can enhance rice nickel tolerance prompted mainly by preventing chloroplast damage as a result of improved N metabolism under excessive nickel contamination (Rizwan et al., 2019). However, the role of H_2_S as a signaling molecule in plants is still not fully understood despite the fact that the release of H_2_S has been demonstrated in many plant species. Notably, desulfhydrases (H_2_S-releasing enzymes) have functionally been endorsed as key H_2_S volatile in plants (Riemenschneider et al., 2005b). In another case, enhanced L-Cys desulfhydrase (LCD) activity under biotic stress further reaffirms its significant potency as an adaptive defense approach under stress agriculture (Rausch and Wachter, 2005). H_2_S was also involved in a promotional role of superior root organogenesis in *Ipomoea batatas*, *Salix matsudana*, and *Glycine max* L. (Zhang et al., 2009).

The antioxidant enzymes are another type of stress mediator in plants, which are often activated as a key defense response after recognition of given stressor, including heavy metals (Zhang et al., 2010a,b). Foliar application of sodium hydrosulfide (NaHS), an H_2_S donor, led to an upscale induction response of different antioxidant enzymes and reduced the concentration of H_2_O_2_ in wheat seedlings to enhance resistance against heat stress. Also, Zhang et al. (2011) emphasized that H_2_S can have an effective role in plant protection against different types of oxidative stress. Water-soluble antioxidants, such as ascorbic acid and glutathione, were expressed at enhanced level upon fumigation with H_2_S, which consequently delivered higher water stress tolerance in wheat plants (Shan et al., 2011). As NaHS is characterized as key antioxidant inducer; therefore, plant growth promotive activities including root organogenesis (Zhang et al., 2009), stomatal regulation (Lisjak et al., 2010), and seed germination (Zhang et al., 2010a,b) were substantially improved in response to heavy metal stresses. Interestingly, exogenously applied H_2_S (100 ppb) has shown striking effect on plant growth improvement of beet, alfalfa, and lettuce (Thompson and Kats, 1978). Furthermore, H_2_S foliar spraying has also improved the accumulation of vital nutrients in plant (Wang et al., 2012).

As a secondary messenger, nitric oxide and H_2_S can collectively trigger signal transduction resulting in higher intracellular buildup of these molecules, which are indeed a plant’s necessity to cope with metal-induced oxidative stress. In this way, use of H_2_S with nitric oxide could exert a regulatory response to transporters and antioxidant systems to alleviate phytotoxic effects of heavy metals (Li et al., 2012; Wang et al., 2019). Considering all the background information about the role of H_2_S in biological system and, most importantly, its interaction with crop plants under stress agriculture, we aimed to get an advanced overview of H_2_S-related growth promotive effects on crop plants. Soil contamination by heavy metals imposes greater ecological concern because of lack of plant cover and resultant land degradation. However, use of stress alleviators, such as H_2_S, along with suitable crop plant holds a great potential for the successful restoration of these contaminated soils. We also focus on how the use of H_2_S and other precursor compounds interacts with plants to counteract heavy metal toxicity. Moreover, our discussion also focuses H_2_S-mediated response mechanisms exhibited by plants toward heavy metal toxicity.

## Chemistry of H_2_S

H_2_S is a weak acid with good water solubility and commonly exists in neutral molecular form (H_2_S). Although HS^–^ is a major ionic form of H_2_S involved in most of its biological reactions, S^2–^ also exists in minor concentration due to higher dissociation constant for second ionization (Filipovic et al., 2018). Despite being a highly water-soluble compound, H_2_S tends to be unstable under natural conditions as it slowly oxidizes to elemental sulfur having a weak solubility in aqueous solution. Moreover, the volatile nature of H_2_S exacerbates its experimental use in the environment. For instance, nearly half-dose of H_2_S could be lost in 5 min from open cell culture wells (DeLeon et al., 2012). Consequently, H_2_S handling imposes greater challenge of its precise measurement under field conditions (Wang et al., 2014; Peng and Xian, 2015).

$$H_{2}\,S⇌{\mathrm{HS}}^{-}+H^{+}⇌S^{2-}+2\,H^{+}\,\left(\mathrm{in}\,\mathrm{aqueous}\,\mathrm{solution}\right)$$

## Occurrence and Biosynthesis of H_2_S in Plants

There has been a proposition long ago that plants can produce and release H_2_S themselves, particularly when exposed to external sulfur (S) stimuli, i.e., Cys, sulfate, sulfite, or SO_2_ (Wilson et al., 1978; Sekiya et al., 1982a,b). This was thought to be a mechanism for regulating sulfur homeostasis (Calderwood and Kopriva, 2014). However, mechanistic understanding of H_2_S generation in plants and its interaction with other cellular components remains elusive. In higher plants, H_2_S biosynthesis pathway emerges in different subcellular compartments, where main enzymes linked to sulfur metabolism have the potential to initiate H_2_S biogenesis (Corpas et al., 2019b; Chen et al., 2020). In plants, most common enzymes involved in the H_2_S biosynthesis enzymes include LCD, D-Cys desulfhydrase (DCD), l-3-cyanoalaninesynthase, sulfite reductase, and Cys synthase (Yamasaki and Cohen, 2016). Among various plant cellular organelles, chloroplast serves as the major H_2_S production site due to localization of sulfite reductase enzyme, which catalyzes the reduction of sulfite to sulfide during sulfate assimilation pathway. In addition to this, cytosol can also generate H_2_S by the action of DCD and LCD, accompanied by ammonia and pyruvate production. In chloroplast, sulfide concentration is two times greater than that found into the cytosol (Krueger et al., 2009). However, this sulfide is dissociated into its ionized forms due to the basic physiological conditions and therefore unable to pass through the membranes to the cytosol (Kabil and Banerjee, 2010). In mitochondria, the synthesis of H_2_S can be regulated by β-cyanoalanine synthase (a pyridoxal phosphate-dependent enzyme), which transforms both cyanide and L-Cys into β-cyanoalanine and H_2_S in order to degrade toxic cyanogen (Gotor et al., 2019). Recently, Corpas et al. (2019a) also provided an evidence of H_2_S generation in the peroxisomes of *Arabidopsis*; however, it is still unclear whether its generation pathway is endogenous or recruited from other cellular compartments (e.g., cytosol). Additionally, the expression of plant cellular proteins [L-Cys desulfurase such as *O*-acetylserine thiol lyase (OAS-TL) and Nifs-like proteins] also processes the H_2_S synthesis in different cellular organelles (Gotor et al., 2019). These enzymes, with their varied expression, are therefore involved in controlling the production of H_2_S across various cellular compartments of the plant. On the other hand, occurrence of H_2_S in these cellular organelles with strong lipophilic characteristic promotes its translocation in the lipid bilayer of cell membranes (Cuevasanta et al., 2017).

H_2_S is a flammable-toxic gaseous molecule that is often distinguished by stinky rotten eggs smell. It has also shown strong concentration dependent affinity for reactions as it can disrupt mitochondrial cytochrome activity and even reduce mitochondrial respiration (Mancardi et al., 2009). Surprisingly, research in recent years has unraveled the significance of H_2_S as a gasotransmitter that promotes plant growth and development at various stages of plant life cycle (Xuan et al., 2020). Other than algae, fungi, and few prokaryotes, plants are known to take leverage of taking up the naturally occurring sulfate (SO_4_^2–^) source of S from soil and incorporate it into organic forms (Takahashi et al., 2011). In S assimilation process, sulfate taken up by plant roots is initially reduced to H_2_S by catalytic activity of adenosine 5′-phosphosulfate reductase and sulfite reductase and eventually transformed into Cys via O-OAS-TL. Therefore, H_2_S is an extremely important intermediate in the thiometabolism pathway.

**FIGURE 1:**
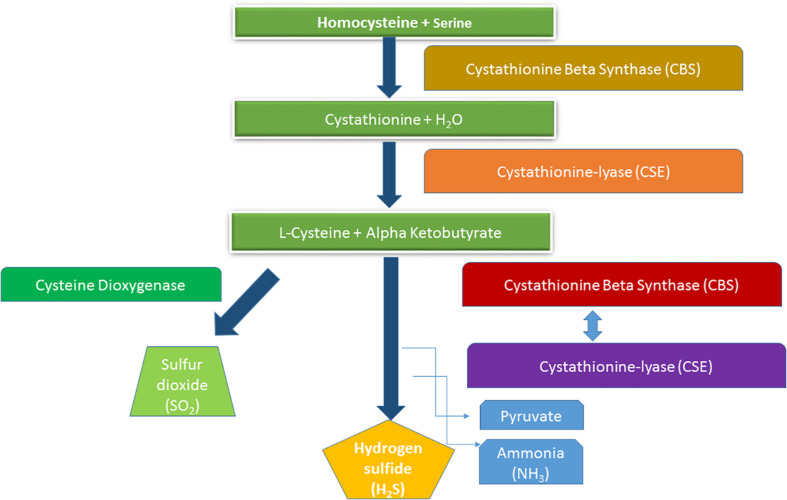
Hydrogen sulfide production through homocysteine metabolism.

The use of H_2_S as signaling molecule has now become very common; thus, basic mechanism of its functional activities has been decrypted (Mustafa et al., 2009; Aroca et al., 2015). Some recent proteomic analyses have described a new posttranslational modification of proteins, where reactive Cys residues (as an H_2_S signal) can modify protein function by converting the thiol group (-SH) into a persulfide group (-SSH) known as persulfidation. In most cases, persulfide adducts exhibit higher nucleophilicity relative to the thiol group, and as a result, modified Cys displayed highly complex reactivity (Paul and Snyder, 2012). This might be the rationale of widespread persulfidation in nature, which largely affects protein over O_2_ and N species (Ida et al., 2014) (Figure 1). Overall, complex functional interactions of H_2_S based on its donor, concentration gradient, and plant section tend to describe actions of specific protein after translational modification.

The nature of H_2_S-mediated specific cellular modifications still lacks clarity, because thermodynamic reaction involving H_2_S and a thiol is unfavorable. Sulfane sulfur is a sulfur atom that has the peculiar ability to bind reversibly to other sulfur atoms to form hydropersulfides (R-S-SH) and polysulfides (-S-Sn-S-). These polysulfides tend to be far more efficient in persulfidation, as they are more nucleophilic than H_2_S (Toohey, 2011). Recently, new molecular weight persulfides were identified as possible mediator of sulfide signaling. In this relation, Cys-persulfide (Cys-SSH), glutathione persulfide, and its persulfurated species Cys-SSnH and GSSnH have been designated as redox regulators (Kasamatsu et al., 2016; Kimura et al., 2017). Recently, the endogenous Cys-SSH production synthetized by prokaryotic and mammalian cysteinyl-tRNA synthetases using L-Cys as substrate has been described. The Cys polysulfides bound to tRNA are incorporated into polypeptides that are synthesized *de novo* in the ribosomes, suggesting that these enzymes are the principal Cys persulfide synthases *in vivo* (Akaike et al., 2017).

## Promotive Role of H_2_S in Regulating Plant Heavy Metal Stress

Of various abiotic stresses in environment, heavy metal-triggered stress always has very serious repercussions for plant growth and productivity. Like other abiotic stress, heavy metal stress is also associated with unregulated overproduction of ROS, which can influence plant metabolism and physiological activities by inflicting range of oxidative stress damages. Among the signaling molecules, H_2_S is now an established regulator of growth in plants exposed to plethora of abiotic stresses, including heavy metal stress (Rather et al., 2020).

## Exogenous H_2_S Application: a Precursor of Heavy Metal Stress Alleviation in Plants

Given a suite of heavy metal mitigation approaches competing for an effective stress management in agriculture, it is extremely pertinent to consider only those measures that can provide plant benefits only in an eco-friendly way. Although H_2_S is toxic for many living organisms as evidenced by mitochondrion inhibition (Wang et al., 2019), its central role in key plant physiological functions and therapeutic use in various human ailments have been well developed in recent times (Aroca et al., 2020). The H_2_S in gaseous form is the simplest method of its usage in the laboratory; however, it is not realistic practice due to non-targeted ecotoxicological consequences both for the humans and environment (Rubright et al., 2017). In most cases, NaHS and/or sodium sulfide (Na_2_S) are used as H_2_S donor molecules specifically due to their higher dissolution, resulting in a short albeit sustained pulse of H_2_S (Table 1).

**TABLE 1:** Effects of exogenous application of different H_2_S sources on plant growth regulations under heavy metals stress.

H_2_S source	H_2_S conc.	Plant species	Experiment	Duration/growth stage	Heavy metal/source	Exposure level	Tolerance mechanisms involved	References
Sodium hydrosulfide (NaHS)	100 μM	Alfalfa (*Medicago sativa* L.)	Potted soil	60 days/young plant	Lead, cadmium	217 mg kg^–1^ of Pb 4.95 mg kg^–1^ of Cd	Upscaled antioxidant activity (POD, CAT, APX, SOD) and reduced oxidative damage (MDA, H_2_O_2_, O^–^_2_) decreased the absorption of metal ions	Fang et al., 2020
Sodium hydrosulfide (NaHS)	100 μM	Zucchini (*Cucurbita pepo* L.)	Potted soil	14 days/young seedlings	Nickel: Ni (NO_3_)_2_	50 mg of Ni	Decreased metal ions accumulation, restored ionic homeostasis and averted oxidative membrane damages by upregulation of phenolic and flavonoid metabolites	Valivand and Amooaghaie, 2020
Sodium sulfite (Na_2_SO_3_)	0.5 mM	Foxtail millet (*Setaria italica* L.)	Hydroponic culture	12 days/young seedlings	Cadmium	100 μM	Suppressed nitrate reductase and nitric oxide synthase–dependent endogenous nitric oxide (NO), which further enhanced the enzyme activities, i.e., SOD and POD, leading to the rescue of Cd related root growth inhibition	Han et al., 2020
Sodium hydrosulfide (NaHS)	200 μM	Cauliflower (*Brassica oleracea* L.)	Potted soil	42 days/transplants	Chromium: K_2_Cr_2_O_7_	10–100 μM	Restricted oxidative stress damages (EL, MDA, H_2_O_2_), increased antioxidant activity (SOD, CAT, APX, POD) in leaves and root tissues	Ahmad et al., 2020
Sodium hydrosulfide (NaHS)	150 μM	Woad plant (*Isatis indigotica* L.)	Hydroponic culture	28 days/young seedlings	Cadmium	22.5 μM	Decreased intracellular metal ion toxicity and induced production of metallothioneins (metal-binding protein) restricting root-to-shoot Cd translocation	Jia et al., 2020
Sodium hydrosulfide (NaHS)	0.2 mM	Cauliflower (*Brassica oleracea* L.)	Hydroponic culture	24 days/young seedlings	Lead: Pb (CH_3_COO)_2_⋅3H_2_O	0.5 mM	Scavenging of ROS through antioxidant activity and Pb ion chelation by non-protein thiol and total glutathione	Chen et al., 2018
Sodium hydrosulfide (NaHS)	0.2 mM	Pepper (*Capsicum annuum* L.)	Sand, peat, and perlite mixed pots	70 days/mature plants	Zinc: ZnSO_4_⋅7H_2_O	O.5 mM	Reduced metal ions uptake, improved N, P, and Fe uptake and promoted antioxidant activities for limiting membrane oxidative stress damage	Kaya et al., 2018
Sodium hydrosulfide (NaHS)	500 μM	Maize (*Zea mays* L.)	Hydroponic culture	19 days/young seedlings	Chromium	200 μM	Alleviated Cr toxicity by reducing the production of cyto-toxic methylglyoxal via glutathione-S-transferase (GST) detoxification	Kharbech et al., 2020
Sodium hydrosulfide (NaHS)	50 μM	Oilseed rape (*Brassica napus* L.)	Hydroponic culture	18 days/young seedlings	Cadmium: CdCl_2_	20 μM	Prompted higher L-cysteine desulfhydrase (LCD) for S-metabolism, minimized Cd translocation to shoots–leaves and prevented chlorosis	Yu et al., 2019
Sodium hydrosulfide (NaHS)	100 μM	Coriander (*Coriandrum sativum* L.)	Hydroponic culture	18 days/young seedlings	Copper: CuSO_4_	100 μM	Reduced electrolyte leakage by regulating antioxidant enzyme activity via ascorbate–glutathione cycle	Karam and Keramat, 2017
Sodium hydrosulfide (NaHS)	200 μM	Black night shade (*Solanum nigrum* L.)	Hydroponic culture	21 days/young seedlings	Zinc: ZnCl_2_	400 μM	Reduced free cytosolic metal content in roots by upregulated metallothioneins mediated Zn-chelation and antioxidant response mechanism	Liu et al., 2016
Sodium hydrosulfide (NaHS)	0.8 mM	Wheat (*Triticum aestivum* L.)	Plastic trays/sand-vermiculite	14 days/young seedlings	Copper: CuSO_4_	100 μM	Ascorbate and glutathione metabolism minimized Cu toxicity by reducing malondialdehyde content and limiting electrolyte leakage	Shan et al., 2012
Sodium hydrosulfide (NaHS)	200 μM	Cotton (*Gossypium hirsutum* L.)	Hydroponic culture	28 days/young seedlings	Lead: Pb (NO_3_)_2_	50 μM	Improved photosynthetic pigmentation, enhanced antioxidant activities and eventually decreasing malondialdehyde (MDA), electrolyte leakage, and H_2_O_2_ production	Bharwana et al., 2014
Sodium hydrosulfide (NaHS)	100 μM	Pea (*Pisum sativum* L.)	Hydroponic culture	30 days/young seedlings	Arsenate: Na_2_HAsO_4_ × 7H_2_O	50 μM	Revitalized redox cell status against arsenate toxicity by promoting ascorbate–glutathione metabolism and counteract ROS induced membrane damage	Singh et al., 2015
Sodium hydrosulfide (NaHS)	100 μM	Rice (*Oryza sativa* L.)	Hydroponic culture	35 days/young seedlings	Nickel: NiSO_4_⋅6H_2_0	200 μM	Enhanced chloroplast biogenesis and regulate nitrogen (N) metabolism via enzymes, i.e., nitrate reductase, nitrite reductase, glutamate synthase, glutamate oxaloacetate transaminase, glutamine synthetase, and glutamate pyruvate transaminase, and boost plant tolerance under Ni stress	Rizwan et al., 2019
Sodium hydrosulfide (NaHS)	200 μM	Rice (*Oryza sativa* L.)	Hydroponic culture	28 days/young seedlings	Mercury: HgCl_2_	100 μM	Sequestered metal ions in roots and prevented plant oxidative damages by maintaining low MDA and H_2_O_2_	Chen et al., 2016

*POD, peroxidase; SOD, superoxide dismutase; CAT, catalase; APX, ascorbate peroxidase; MDA, malondialdehyde; H_2_O_2_, hydrogen peroxide, EL, electrolyte leakage.*

Production of ROS, by-product of physiological metabolism, is a typical plant response under abiotic stress including heavy metal stress. Nevertheless, plants vary in their response against the different oxidative stresses linked to ROS in species-cultivar-specific ways. In plants, a robust antioxidative defense system (enzymatic and non-enzymatic) has been evolved to scavenge the excessive ROS accumulation, which can in turn counteract the harmful impacts of oxidative stress. Increasing evidence demonstrated that NaHS treatment can ameliorate and repair oxidative stress caused by heavy metal toxicity (Luo et al., 2020). Exogenous application of H_2_S can suppress the burst of ROS by activating enzymatic and non-enzymatic defense components of ascorbate–glutathione cycle and eventually avert oxidative stress damage to plants.

Legume plants are of considerable significance for the remediation of heavy metal-polluted soil due to the unique symbiotic assemblage of N-fixing bacteria (rhizobia) with leguminous host plant (Reichman, 2007; Shen et al., 2019). In alfalfa, exogenous application of NaHS (100 μM) has mitigated the compounding effects of dual metal stress (Pb/Cd) on legume-rhizobium symbiosis (Table 1; Fang et al., 2020). Application of H_2_S donor molecule boosted up the survival rate of rhizobia by enhancing soil enzyme activity, facilitating nutrient transformations, and shifting both composition and diversity of soil microbial population. This study concludes that H_2_S-mediated symbiosis has resulted in development of greater plant resistance to metal-induced toxicity as evident by increased antioxidant enzyme activity and reduced metal ion absorption. Similar to these findings, Mostofa et al. (2015) investigated a rice–cadmium interaction model, where H_2_S provided further evidence of being an efficient growth regulator to mitigate Cd-related growth suppression and reduction in biomass. Moreover, rice growth revitalization performance was primarily triggered by a three-way Cd alleviation process, including low Cd uptake/accumulation, mineral nutrient upregulation, and photosynthetic functions and timely induction of antioxidant response.

Heavy metals, such as Ni, are known to cause disruption in the absorption and utilization of key mineral elements in plants (Sharma and Dhiman, 2013). Importantly, Ni metal ions demonstrate a strong competitive affinity for bivalent cations, i.e., Ca, Mg, Mn, Fe, Zn, which could reduce uptake of these essential elements and obstruct normal plant growth and development (Ahmad et al., 2011). In hydroponic culture, NaHS (100 μM)-treated young seedlings of Zucchini plant showed a decrease in Ni accumulation by reviving essential mineral homeostasis (Valivand and Amooaghaie, 2020). Furthermore, this study validates the role of H_2_S in osmotic adjustment, as indicated by the proline and sugar content of plant exposed to heavy metal stress. Clearly, NaHS facilitated an increase in flavonoid and phenolic secretions likely to reduce the oxidative damage due to ROS scavenging, which in turn led to the improved Ni tolerance in *Zucchini* seedlings.

Phenolic and flavonoid are not the solitary metabolites those are exuded by heavy metal–stressed plants. Ahmad et al. (2020) demonstrated that NaHS treatment can upregulate antioxidant enzyme activity in Cr-exposed cauliflower seedlings, which is central to the amelioration of Cr-related oxidative damages, as well as reduction in metal ion translocation, to aerial plant parts. Root growth is an important predictor of plant productivity in agriculture, while impaired root growth has often been distinguished as one of the most common and earliest symptoms of plant exposure to metal toxicity (Liu et al., 2018). In foxtail millet, Cd-induced root growth inhibition was reversed by an SO_2_ derivative compound Na_2_SO_3_ (Han et al., 2020). This study concludes the existence of crosstalk between SO_2_ and nitric oxide (NO) for nitrate reductase (NR)–nitric oxide synthase-dependent endogenous NO signaling that prompted an upregulated antioxidant enzyme activity and suppressed genes associated with the Cd uptake (SiNRAMP1, SiNRAMP6, SiIRT1, and SiIRT2).

Plant cell wall is a first architectural barrier to avert transmembrane movements of toxic materials, such as heavy metals (Krzesłowska, 2011), as it can allow cellular compartmentation of the metal ions and reduce the phytotoxic effects of heavy metal exposure (Lai, 2015). Metal-tolerant proteins such as metallothionein and phytochelatins are crucial metal-binding ligand that regulates cationic homeostasis of plant cell wall (Yu et al., 2018; Zhi et al., 2020). In Woad plants, Jia et al. (2020) identified strict connection between Cd chelation and S metabolic products based on the weakening of metal ion translocation from root to shoot. The study confirmed that NaHS stimulated the endogenous metal binding proteins, i.e., metallothionein 1A and phytochelatins, which in turn promoted Cd accumulation in the cell wall by modifying its contents, thus reducing intracellular metal ion mobility for detoxification. Previously, Jia et al. (2016) established an intertwining effect of H_2_S and Cys in *A. thaliana* L. They found that sulfur metabolism has key role to play in the growth and development of plants exposed to Cd toxicity. Collectively, plant stress alleviators such as H_2_S and Cys have been shown to resurrect root growth, as well as to increase plant Cd tolerance via S metabolite feedback loop. An active synergy between H_2_S and proline pulls together millet plant from negative effects of Cd toxicity, as evidenced by induced Cd tolerance and stimulated biomass production (Tian et al., 2016). Apart from higher proline accumulation, as well as proline dehydrogenase (PDH) and proline-5-carboxylate reductase (P5CR) activities, the transcript levels of PDH and P5CR were also enhanced after H_2_S treatment.

Also, Yu et al. (2019) have shown that exogenous NaHS application can increase Cd retention into the root cell wall of oilseed rape by stimulating LCD activity. Furthermore, NaHS led to increase in cellular pectin, and root methylesterase activity validates higher metal-binding capacity of root cell that repressed Cd translocation to aerial plant sections. In another study, downregulated metal homeostasis and uptake were recorded when young seedlings of black night shade plant were treated with NaHS under Zn stress (Liu et al., 2016). It has been noted that expression of the metallothionein was increased, leading to an improvement in plant Zn tolerance as shown by the chelation of excessive Zn in the cytoplasm. Moreover, elevated expression of antioxidant enzyme, CAT2, also prevented oxidative stress damages in metal-stressed plants as a result of H_2_S treatment.

Seed viability is one of the crucial determinants of healthy plant growth and increased production. Seed germination and its emergence and subsequent seedling establishment contribute proportionally to achieve sustainability, growth, and productivity. Exogenous NaHS application has improved both seed germination and seedling emergence in cauliflower by scavenging Pb-induced ROS (Chen et al., 2018). Interestingly, H_2_S-led exhibition of plant protection against Pb stress was comparable to ROS scavengers, i.e., 4,5-dihydroxy-1,3-benzene disulfonic acid and N, N′-dimethylthiourea. They concluded that non-enzymatic antioxidants, such as non-protein thiol and total glutathione, were upregulated by H_2_S and improved Pb tolerance by ROS-scavenging and/or directly chelating metal ions. Plant exposure to metal ions often represses the activity of transporters, resulting in ionic imbalance and reduced nutrients assimilation in plants (Vaculík et al., 2020). In Zn-exposed pepper plants, exogenous NaHS treatment reduced Zn plant accumulation and enhanced the absorption of key mineral elements, i.e., Fe, N, P (Kaya et al., 2020). Moreover, mitigating effects of NaHS have been further up-scaled by antioxidant activity and osmotic adjustment to minimize membrane oxidative damage. Methylglyoxal (MG), a cytotoxic metabolic by-product of glycolytic pathways, usually interacts with macromolecules to trigger protein inactivation and induces oxidative damages to plant under abiotic stress, such as heavy metals (Hoque et al., 2016; Bhuyan et al., 2020). In maize, Cr tolerance of young seedlings was linked to the suppression of NADPH oxidase activity, resulting in restricted ROS accumulation following exogenous application of NaHS (Kharbech et al., 2020). This study illustrated the potential function of glutathione in minimizing Cr toxicity by reducing MG content while preserving glutathione–ascorbate homeostasis for additional S metabolism, as demonstrated by the activity of glutathione S-transferase and reductase enzymes. Similarly, NaHS pretreatment also reduced the lipid peroxidation and electrolyte leakage in coriander seedling exposed to Cu toxicity (Karam and Keramat, 2017). These results further substantiate that NaHS-led changes in endogenous H_2_S were presumably involved in the prevention of oxidative damages via cellular ascorbate–glutathione cycle. Similarly, mitigation of Cu toxicity in wheat has been linked to H_2_S-related ascorbate–glutathione cycle (Shan et al., 2012). Moreover, decrease in lipid peroxidation and electrolyte leakage further implies a systemic defense response activated by NaHS application.

Photosynthesis is a key physiological process for plant productivity and directly provides energy for plant growth. Photosynthetic pigments are very sensitive to various abiotic stress including heavy metals, which can negatively affect rate of photosynthesis via chlorophyll and carotenoid degradation (Amari et al., 2017). RuBISCO (a multi-meric photosynthetic enzyme), reflects potential for plant productivity and the efficiency of resource use by its net C assimilation rate. There is some substantial evidence in literature that described about H_2_S role of being a key regulator of plant photosynthetic apparatus (Carmo-Silva et al., 2015; Cummins et al., 2018). In spinach, Chen et al. (2011) found that NaHS-treated plants had significant increase in chlorophyll content alongside higher soluble protein content and biomass yield. This possibly highlights the significance of RuBISCO activity, which promotes chloroplast biogenesis, photosynthetic enzyme expression, and thiol redox alterations. Also, Bharwana et al. (2014) reported that NaHS application could regulate the photosynthetic activity of cotton seedlings under Pb toxicity. They concluded that higher photosynthesis is obviously a defense mechanism intended to minimize metal toxicity by accelerating rate of photosynthesis, which ultimately contribute to cope with predictable oxidative stress. Further results showed that H_2_S also promoted the reversal of electrolyte leakage in cotton seedlings caused by Pb toxicity, as shown by reduced H_2_O_2_ and MDA contents. In another study, Rizwan et al. (2019) reported NaHS-induced upregulation of chloroplast biogenesis and N metabolism in rice plant exposed to Ni stress. The NaHS application has been found to increase the activities of various N-related enzymes, i.e., NR, nitrite reductase, glutamate synthase, glutamate oxaloacetate transaminase, glutamine synthetase, and glutamate pyruvate transaminase. Furthermore, key involvement of H_2_S in Ni stress regulation of rice plant was also validated by the expression of genes abundance associated with N metabolism. Also, Chen et al. (2016) revealed the molecular basis of rice stress adaptation against mercury (Hg) contamination. In their study, H_2_S pretreatment extended membrane transcriptional expression of bZIP60 and OsMT-1, which were involved in Hg localization in roots. In addition, Hg-related plant membrane damages were attenuated by scavenging ROS and downregulation of H_2_O_2_ and MDA, which eventually led to growth promotion of rice seedlings. The redox status of plant cell can be disrupted by accumulation of ROS associated with metal toxicity, resulting in a cascade of retarded physiological and morphological functions (Schutzendubel and Polle, 2002). In pea seedlings, H_2_S application restored the cellular redox status of pea suffering from arsenate toxicity (Singh et al., 2015). It appears that H_2_S-mediated recovery of ascorbate–glutathione enzymes pool was a pivotal contributor to plant defense, as depicted by suppression of oxygen free radicals and membrane damage.

## Stress Alleviators and Endogenous H_2_S: a Combating Tool Toward Metal Detoxification

In some recent reports, enrolment of different plant growth regulators has also unveiled endogenous H_2_S synthesis in plant that eventually provide a protective role in crop plants under stress (Table 2). NO has been deemed to be an important signaling compound that can stimulate plant growth and development in agriculture. A plethora of studies has investigated the key involvement of NO in activating plant defense response to heavy metal stress (Bai et al., 2014; Rizwan et al., 2018; Hu et al., 2019; Sharma et al., 2020a). A strong synergic association between exogenously applied NO and resultant endogenous synthesis of H_2_S was involved in enhanced Cr resistance of tomato seedlings (Alamri et al., 2020). It appears that increased S assimilation and related enzyme metabolic activities have mitigated the depressing effect of metal ions in both cellular and molecular levels. Salicylic acid (SA), a phenolic compound, has been known to control a broad range of physiological and biochemical functions in plants to combat stressful conditions, including heavy metals (Hasanuzzaman et al., 2019; Sharma et al., 2020b). The exogenous application of Na_2_SiO_3_, a silicon derivative compound, endorsed a functional relationship between NO and H_2_S in regulating the Cd stress of pepper plant (Kaya et al., 2020). It was evident that upregulation of endogenous NO and H_2_S was central to metal stress alleviation as confirmed by lower Cd content of leaves. In addition, improvement in plant antioxidant activities, nutrients uptake (e.g., Ca, K), photosynthesis activity, and water relations upscaled the plant metal tolerance. In pepper experiment, SA prompted 66% higher buildup of endogenous H_2_S in Pb-exposed leaves over untreated control (Kaya, 2020). It was further elucidated that efficient crosstalk between SA and H_2_S averted induced phytotoxicity effect by minimizing Cd accumulation in leaves via upregulated metabolisms of the enzymes involved in ascorbate–glutathione cycle. Furthermore, SA and H_2_S augmented leaf relative water, water potential, and proline content, which has key contribution in restoring the phenotypic appearance of Pb-stressed plants. In another study with maize exposed to Pb stress, Zanganeh et al. (2018) pointed out the synergic effect of SA and H_2_S, which contributed to the relegation of metal-induced phytotoxicity by augmenting glycine-betaine and NO signaling. It was found that SA and NO signaling play an instrumental role in plant growth regulation as they boosted the S-assimilation via arginine–methionine metabolism and thus prevented Pb-induced oxidative stress injury in plants.

**TABLE 2:** Use of different stress alleviators and their interactions with endogenous H_2_S for higher metal tolerance in plants.

Stress alleviator	Exogenous H_2_S	Plant species	Experiment	Duration/growth stage	Heavy metal/source	Exposure level	Endogenous H_2_S	Stress alleviating symptoms	References
Silicon (Na_2_SiO_3_) 2.0 mM	NO	Pepper (*Capsicum annuum* L.)	Potted soil	35 days/young seedlings	Cadmium: CdCl_2_	0.1 mM	Upregulated ∼10 μmol g^–1^ fresh weight	Reduced leaf Cd content and oxidative stress Improved K and Ca uptake	Kaya et al., 2020
Nitric oxide (NO) 50 μM	NO	Tomato (*Solanum lycopersicum* L.)	Pot filled with vermiculite-perlite	21 days/young seedlings	Chromium: K_2_Cr_2_O_7_	100 μM Cr (VI)	Upregulated ∼98 nmol g^–1^ fresh weight	Increased S assimilation and kinked enzyme metabolism averted DNA and oxidative damages.	Alamri et al., 2020
Thiamine (THI) 50 mgL^–1^	0.2 mM sodium hydrosulfide (NaHS)	Strawberry (*Fragaria × ananassa* Duch)	Hydroponic culture	28 days	Cadmium: CdCl_2_	0.1 mM	Upregulated ∼15 μmol g^–1^ fresh weight	Upregulated endogenous NO and hydrogen sulfide (H_2_S); Enhanced Ca and K uptake	Kaya and Aslan, 2020
0.5 mM salicylic acid (SA)	0.2 mM sodium hydrosulfide (NaHS)	Pepper (*Capsicum annuum* L.)	Perlite pot	28 days/young seedlings	Lead; PbCl_2_	0.1 mM	Upregulated ∼23 μmol g^–1^ fresh weight	Pb induced phytotoxicity was alleviated by SA + NaHS related upregulation in ascorbate–glutathione cycle	Kaya, 2020
Calcium (CaCl_2_) 15 mM	100 μM sodium hydrosulfide (NaHS)	Zucchini (*Cucurbita pepo* L.)	Hydroponic culture	14 days/young seedlings	Nickel: Ni (NO_3_)_2_	50 mg L^–1^	Upregulated ∼323 nmol g^–1^ fresh weight	Both extracellular (Ca^2+)^ and intracellular (CaM) complex activated crosstalk of Ca^2+^ and H_2_S to revert plant oxidative damages	Valivand et al., 2019
0.5 mM salicylic acid (SA)	0.5 mM sodium hydrosulfide (NaHS	Maize (*Zea mays* L.)	Potted sand–perlite	9 days/young seedlings	Lead: Pb (NO_3_)_2_	2.5 mM	Not detected	Regulated metal toxicity by increasing glycine betaine and NO at the expense of S-amino acids metabolism, i.e., arginine, methionine	Zanganeh et al., 2018
0.5 mM salicylic acid (SA)	NO	Maize (*Zea mays* L.)	Potted sand–perlite	9 days/young seedlings	Lead: Pb (NO_3_)_2_	2.5 mM	Upregulated ∼3.5 μmol g^–1^ fresh weight	Minimized chlorophyll related damages, built-up ascorbic acid glutathione and upregulation of antioxidant enzyme activity	Zanganeh et al., 2019
1 μM methyl jasmonate (MeJA)	50 μM sodium hydrosulfide (NaHS)	Foxtail millet (*Setaria italica* L.)	Hydroponic culture	14 days/young seedlings	Cadmium chloride (CdCl_2_)	200 μM	Upregulated 75–120 nm g^–1^ fresh weight	Alleviated growth retardations by decreasing H_2_O_2_ and malondialdehyde content, also repressed Cd accumulation in seedlings	Tian et al., 2017

Thiamine (THI) is another unique biomolecules that can effectively control plant growth by facilitating the synthesis of carbohydrate, nucleic acids, adenosine triphosphate, and nicotinamide adenine dinucleotide phosphate (Nosaka, 2006). At the same time, it can also activate defensive responses in plants as a non-cofactor (Bettendorff and Wins, 2013; Yusof et al., 2015). Recent work utilizing THI as growth regulators in strawberry transplants exposed to Cd stress has revealed a 1.7-fold increase in endogenous H_2_S levels and displayed enhanced metal tolerance (Kaya and Aslan, 2020). They found that endogenous NO levels have also demonstrated the similar rise following THI treatment. The increase in leaf H_2_S concentration caused the upregulation of MDA and H_2_O_2_, while antioxidant enzyme activities were downregulated to overcome Cd toxicity. Furthermore, endogenous H_2_S improved mechanical stability and physiological functions of strawberry plants by markedly increasing the uptake of key nutrient elements, i.e., calcium and potassium. Although calcium (Ca) is typically an essential macronutrient required for normal plant growth, it can act as a universal messenger to establish systemic defense response and tolerance acquisition under stressful conditions (Jalmi et al., 2018). Several studies confirmed that exogenous Ca application can lead to higher stress tolerance in plants upon heavy metal exposure (Gonzalez et al., 2012; Ahmad et al., 2015; Aziz et al., 2015). According to Valivand et al. (2019), two-sided crosstalk between exogenous Ca and endogenous H_2_S appears to mediate defense response in *Zucchini* plant exposed to Ni stress. The Ca signaling cascade from roots to leaves has been involved in endogenous H_2_S synthesis that contributes toward a systemic acclimation against Ni stress. At the same time, antioxidant enzyme activities and genetic expression of calmodulin (CaM) protein further strengthen the metal ion tolerance of young seedlings, as demonstrated by reduced electrolyte leakage and oxidative injury. This study further supports the fact that both intracellular and extracellular Ca-based complexes are important for the synthesis of endogenous H_2_S to improve plant metal tolerance. Ascorbate–glutathione metabolism involves antioxidant defense system that perceives stress and regulates plant growth by coordinating the activities of detoxification of ROS by its key enzymes–ascorbate peroxidase, monodehydroascorbate reductase, dehydroascorbate reductase, and glutathione reductase (Hasanuzzaman et al., 2017). In maize, Zanganeh et al. (2019) showed that endogenous H_2_S positively regulated early growth of young seedlings via SA-mediated Pb detoxification. Plant exposure to metal ion significantly deteriorated chlorophyll content and restricted nutrients uptake, whereas H_2_S-SA crosstalk was instrumental in reversal of selected plant physiological and biochemical attributes, indicated by higher plant expression of ascorbic acid–glutathione metabolic activities. Methyl jasmonate (MeJA), a volatile derivative of jasmonic acid, serves as main cell signaling molecule mediating various key plant processes and also triggers plant defense in response to wide array of abiotic stresses (Raza et al., 2020). In foxtail millet, exogenous MeJA and NaHS showed an increase in endogenous H_2_S (75–120 nm g FW^–1^) and restore seedling growth under Cd stress (Tian et al., 2017). The results suggest that endogenous H_2_S buildup was a pivotal component of ROS mitigation, thus hindering the accumulation of Cd in young seedlings. In addition, positive interplay between MeJA and H_2_S augmented Cd-induced expression of the homeostasis-related genes (MTP1, MTP12, CAX2, and ZIP4).

## Conclusion and Future Prospective

In agriculture, abiotic stress involving heavy metals contributes to major production losses globally. It is broadly acknowledged that H_2_S can mitigate the abiotic stresses including heavy metal stress. This review provides insight into beneficial aspects of H_2_S on plant biochemical and physiological responses against heavy metal stress. After a thorough review of the available literature, we found that heavy metals inhibit plant growth, which unfortunately disturbs food production and eventually leads to food shortfall. Application of H_2_S has shown effective mitigation of heavy metals related by strengthening the biochemical and physiological functions of plants. Treatment of H_2_S devotedly leads to the enhancement in plant growth, photosynthetic pigments, biomass, nutrient uptake, gas exchange parameters, and antioxidant enzymes of plants.

Taking due account of the above findings and studies, it is strongly concluded that H_2_S treatment effectively reduces the harmful effects of many heavy metals (Al, Cd, B, Pb, Cr, and Cu) by obstructing the accumulation of heavy metals in various plants. Such results strongly indicate that H_2_S treatment could be used effectively as a signal molecule to inhibit the oxidative stress caused by heavy metal contamination. Recently, interaction of certain signal molecules and growth regulators with H_2_S application has been investigated in order to regulate plant growth against heavy metal stress. Nonetheless, future work on other emerging signaling molecules and phytohormones is highly desirable, which can provide us a better understanding of these signaling molecules that affect the concentration of plant hormones and thus control the toxicity of metals in plants. Moreover, further investigations at genomics, transcriptomic, and metabolomics scale are required to explore the particular H_2_S-generated tolerance mechanism in various plants against heavy metal stress. On the other hand, effective, widespread, and ongoing field trials using organic amendments and/or metal-tolerant microbial inoculant can also be tested for their protective role against the exposed contaminants, which can also upscale the effectiveness of H_2_S in heavy metal-contaminated degraded land.

## Author Contributions

All authors listed have made a substantial, direct and intellectual contribution to the work, and approved it for publication.

## Conflict of Interest

The authors declare that the research was conducted in the absence of any commercial or financial relationships that could be construed as a potential conflict of interest.
